# Supplementary material for: Boosting thermoelectric performance of single-walled carbon nanotubes-based films through rational triple treatments
Source: Nat Commun. 2024 Apr 23;15:3426. doi: 10.1038/s41467-024-47417-y (PMC11039726; doi:10.1038/s41467-024-47417-y)
Supplement: Supplementary file 1 — Supplementary Information [file 41467_2024_47417_MOESM1_ESM.pdf]

Supplementary Information

Boosting thermoelectric performance of single-walled carbon nanotubes based films through rational triple treatments

*Yuan-Meng Liu,[1,a] Xiao-Lei Shi,[2,a] Ting Wu,[1] Hao Wu,[1] Yuanqing Mao,[2,3,4] Tianyi Cao,[2] De-Zhuang Wang,[1] Wei-Di Liu,[2] Meng Li,[2] Qingfeng Liu,[1, *] and Zhi-Gang Chen[2, *]*

[1] State Key Laboratory of Materials-Oriented Chemical Engineering, College of Chemical Engineering, Nanjing Tech University, Nanjing 211816, China.

[2] School of Chemistry and Physics, ARC Research Hub in Zero-emission Power Generation for Carbon Neutrality, and Centre for Materials Science, Queensland University of Technology, Brisbane, Queensland 4000, Australia.

[3] School of Mechanical and Mining Engineering, The University of Queensland, Brisbane, Queensland, 4072, Australia.

[4] Department of Physics and Guangdong Provincial Key Laboratory of Computational Science and Material Design, Southern University of Science and Technology, Shenzhen 518055, China.

[a] These authors contributed equally: Yuan-Meng Liu, Xiao-Lei Shi.

Corresponding Author:

* Qingfeng Liu: qfliu@njtech.edu.cn;

* Zhi-Gang Chen: zhigang.chen@qut.edu.au.

Supplementary Table 1. Comparison of strategies and thermoelectric properties of single-walled carbon nanotubes (SWCNTs)-based thin films. Here S-SWCNTs are abbreviated from semiconducting SWCNTs, DDB-F_{72} is abbreviated from 3,5-bis(trifluoromethyl)benzyloxy, PEDOT:PSS is abbreviated from poly(3,4-ethylenedioxythiophene):poly(styrenesulfonate), DMSO is abbreviated from dimethyl sulfoxide, AgTFSI is abbreviated from silver bis(trifluoromethanesulfonyl)imide, PEDOT:PF_6 is abbreviated from poly(3,4-ethylenedioxythiophene):hexafluorophosphate, PFO-Py is abbreviated from Poly[(9,9-dioctylfluorenyl-2,7-diyl)-co-(1,4-benzo-{2,1',3}-thiadiazole)], and PANI is abbreviated from polyaniline.

**Materials**	**Strategy**	σ **(S cm^{-1})**	S **(μV K^{-1})**	$S^2\sigma$ **(μW cm^{-1} K^{-2})**	**Ref.**
SWCNTs	$NaBH_4$-pressing	14356	37. 6	20.29	**This work**
S-SWCNTs	DDB-F_{72}	>1000	≈80	9.17	[1]
PEDOT:PSS/SWCNTs	NaOH	1701	55.6	5.26	[2]
PEDOT:PSS/SWCNTs	DMSO-non-solvent turbulent secondary doping	4717.8	32.6	5.01	[3]
S-SWCNTs	AgTFSI	≈1000	≈60	4.12	[4]
PEDOT:PSS/SWCNTs	$NaBH_4$	1718	49	4.11	[5]
PEDOT:PF_6/SWCNT		~900	~53	2.54	[6]
SWCNTs		680	60	2.45	[7]
S-SWCNTs	PFO-Py/toluene	288	92	2.43	[8]
PANI/SWCNT	DMSO	842.5	~53	2.36	[9]
SWCNTs		1510	38	2.2	[10]
SWCNTs		774.7	50.8	2.07	[11]

Supplementary Table 2. X-ray photoelectron spectroscopy (XPS) characterized the content of atomic in different SWCNTs films. Here "SWCNTs-pressing" indicates the SWCNTs films after cold pressing, "SWCNTs-rolling" indicates the SWCNTs films after rolling, and "SWCNTs-0.1" indicates the SWCNTs films with 0.1 M L^{-1} $NaBH_4$ treatment.

**Atomic content (%)**	**SWCNTs**	**SWCNTs-pressing**	**SWCNTs-rolling**	**SWCNTs-0.1**	**SWCNTs-0.1-pressing**	**SWCNTs-0.1-rolling**
C1*s*	94.2	96.5	94.3	94.9	97. 0	97.5
O1*s*	5.6	3.5	5.7	4.7	2.8	2.5

Supplementary Table 3. X-ray photoelectron spectroscopy (XPS) characterized the position of atomic in different SWCNTs films. Here "SWCNTs-pressing" indicates the SWCNTs films after cold pressing, "SWCNTs-rolling" indicates the SWCNTs films after rolling, and "SWCNTs-0.1" indicates the SWCNTs films with 0.1 M L^{-1} $NaBH_4$ treatment.

**Binding energy(eV)**	**SWCNTs**	**SWCNTs-pressing**	**SWCNTs-rolling**	**SWCNTs-0.1**	**SWCNTs-0.1-pressing**	**SWCNTs-0.1-rolling**
C1*s*	284.8	284.9	284.8	288.1	284.7	284.9
O1*s*	532.2	532.4	532.0	535.6	532.2	532.3

Supplementary Table 4. Comparison of thermoelectric properties of SWCNTs-based devices. Here dPhiz-6 is abbreviated from π-conjugated organic small molecules (π-OSMs) with twisted phenyl rings, PEI is abbreviated from polyethyleneimine, and NDI-T2 is abbreviated from naphthalene-diimide (NDI)-based conjugated polymers.

**p-type materials**	**n-type materials**	**Couple number**	**ΔT (K)**	**Open-circuit voltage (mV)**	**Output power (μW)**	**Output power density (μW cm^{-2})**	**Ref.**
SWCNTs		6	40	9.8	0.86	2996	This work
PEDOT:PSS/SWCNTs		8	20	6.34	0.391	60.9	[5]
PEDOT:PSS/SWCNTs		5	58	7.3	4.416		[3]
SWCNTs/dPhiz-6		5	30	16.5	3.2		[12]
SWCNTs	SWCNT films doped by PEI	3	27.5	5	2.5		[13]
PEDOT:PSS/SWCNTs		6	60	6.6	1.2		[14]
PEDOT:PSS/SWCNTs	SWCNT films doped by PEI	6	50	28.2	0.220		[15]
NDI-T2/SWCNTs		5	20	8.02	0.0272		[16]
SWCNTs/aspartic acid	SWCNT/lysine	5	87	42.3	0.0043		[17]

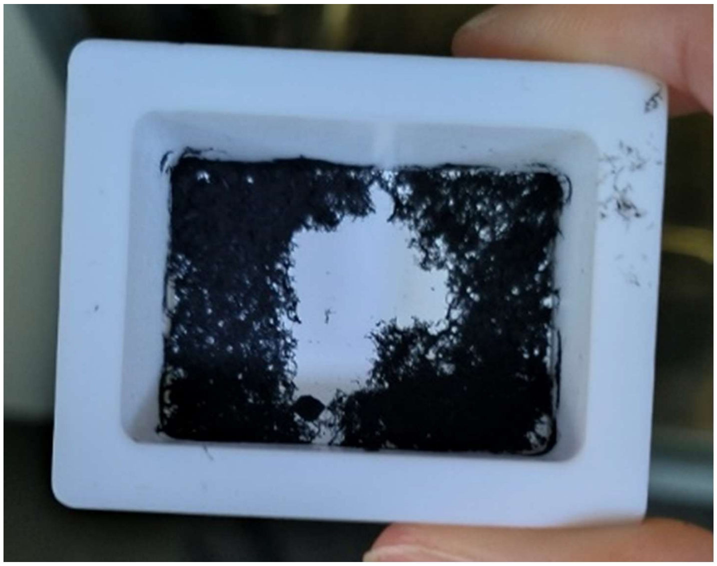

Supplementary Fig. 1 Photograph indicating that single-walled carbon nanotubes (SWCNTs) cannot form a film without ultrasonication.

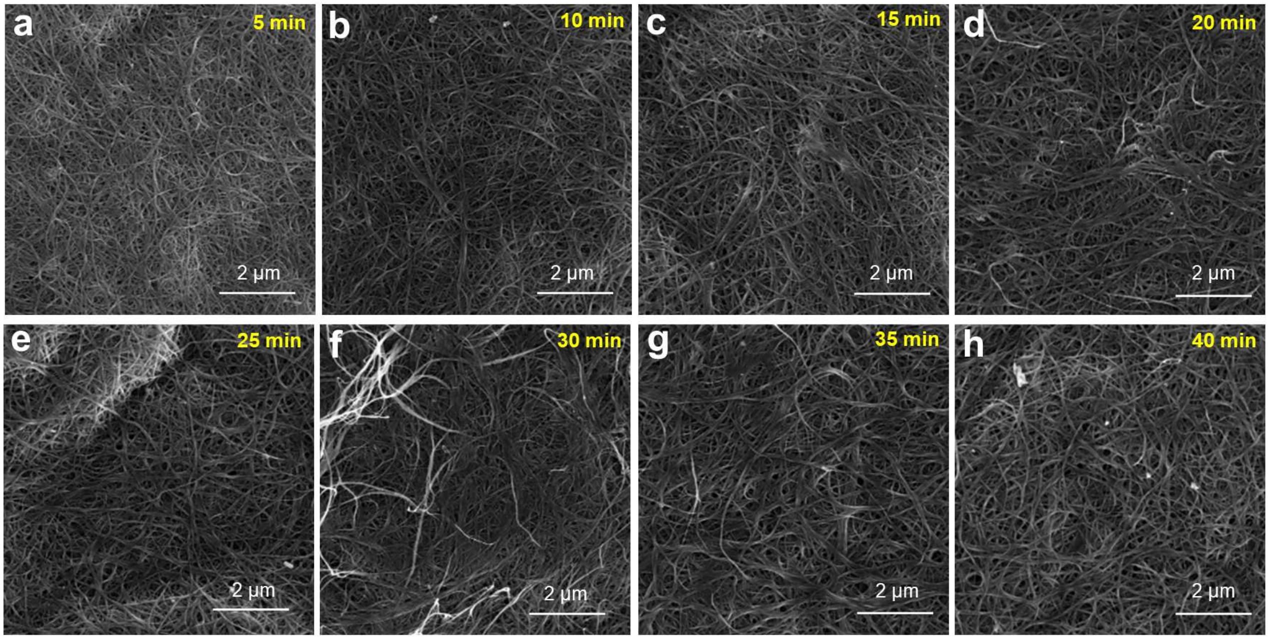

Supplementary Fig. 2 Scanning electron microscopy (SEM) images of SWCNT films by different ultrasonic times. **a** 5 min. **b** 10 min. **c** 15 min. **d** 20 min. **e** 25 min. **f** 30 min. **g** 35 min. **h** 40 min.

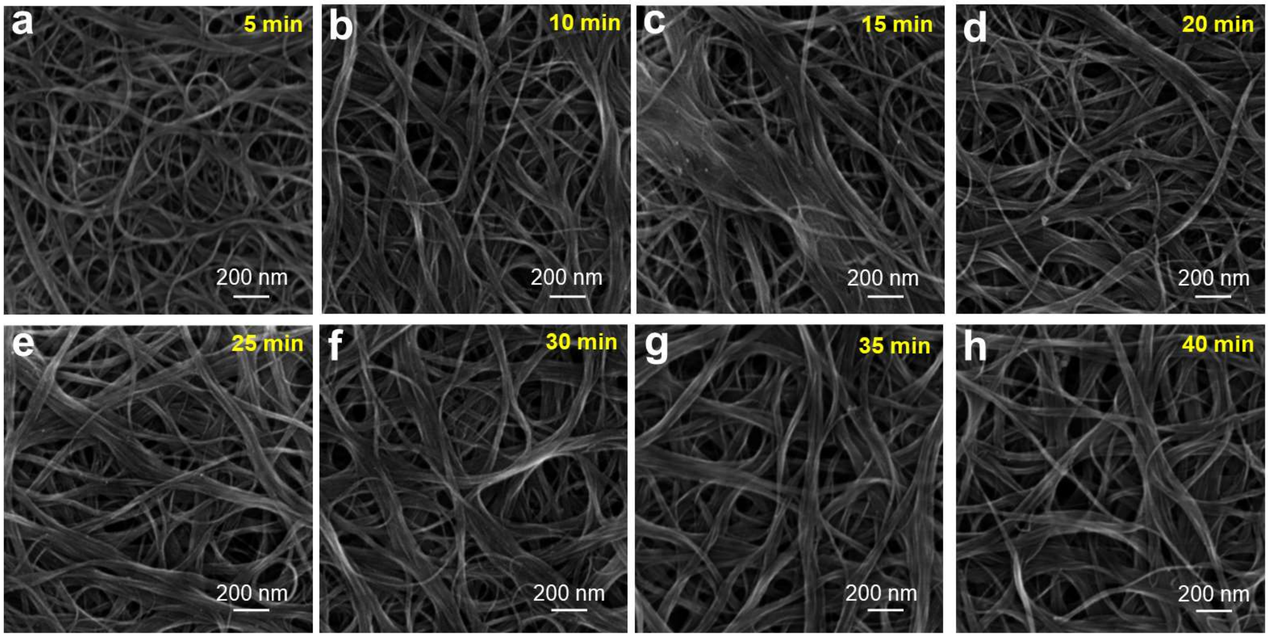

Supplementary Fig. 3 High-magnification SEM images of SWCNT films by different ultrasonic times. **a** 5 min. **b** 10 min. **c** 15 min. **d** 20 min. **e** 25 min. **f** 30 min. **g** 35 min. **h** 40 min.

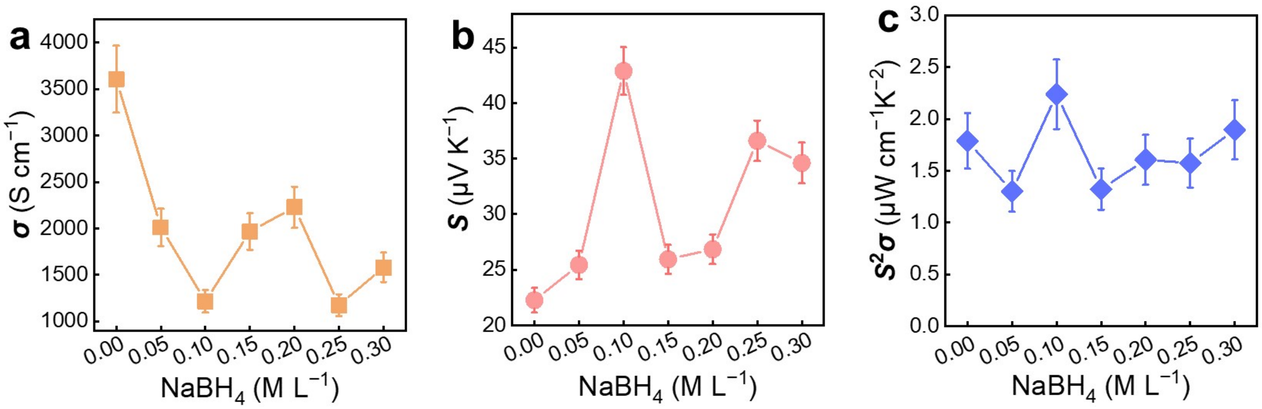

Supplementary Fig. 4 Thermoelectric performance of SWCNT films by different concentrations of $NaBH_4$ treatments. Room-temperature (**a**) electrical conductivity σ, (**b**) Seebeck coefficient S, and (**c**) power factor $S^2\sigma$ of SWCNT films as a function of $NaBH_4$ concentration. Here M is abbreviated from "mol".

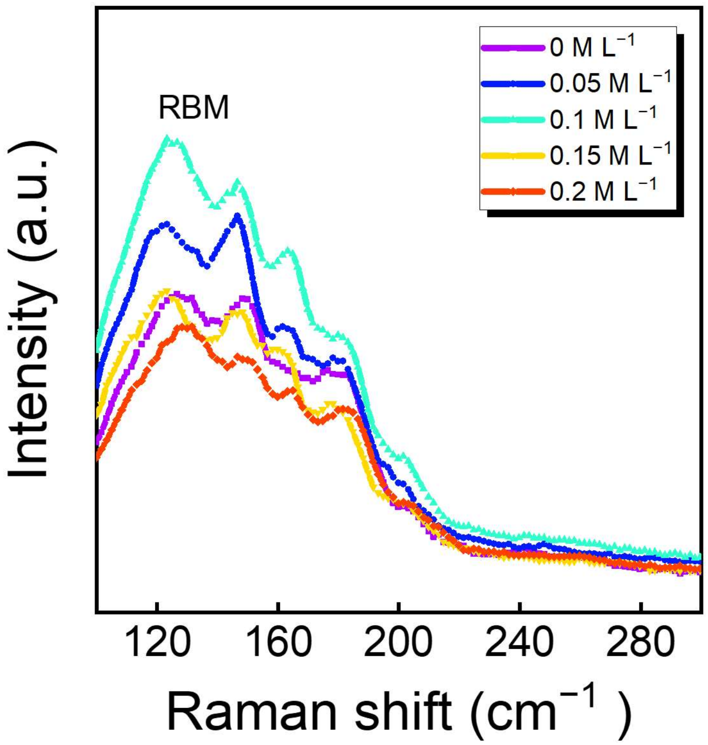

Supplementary Fig. 5 Raman spectra of SWCNT films treated with different concentrations of $NaBH_4$. Here RBM is abbreviated from radial breathing mode.

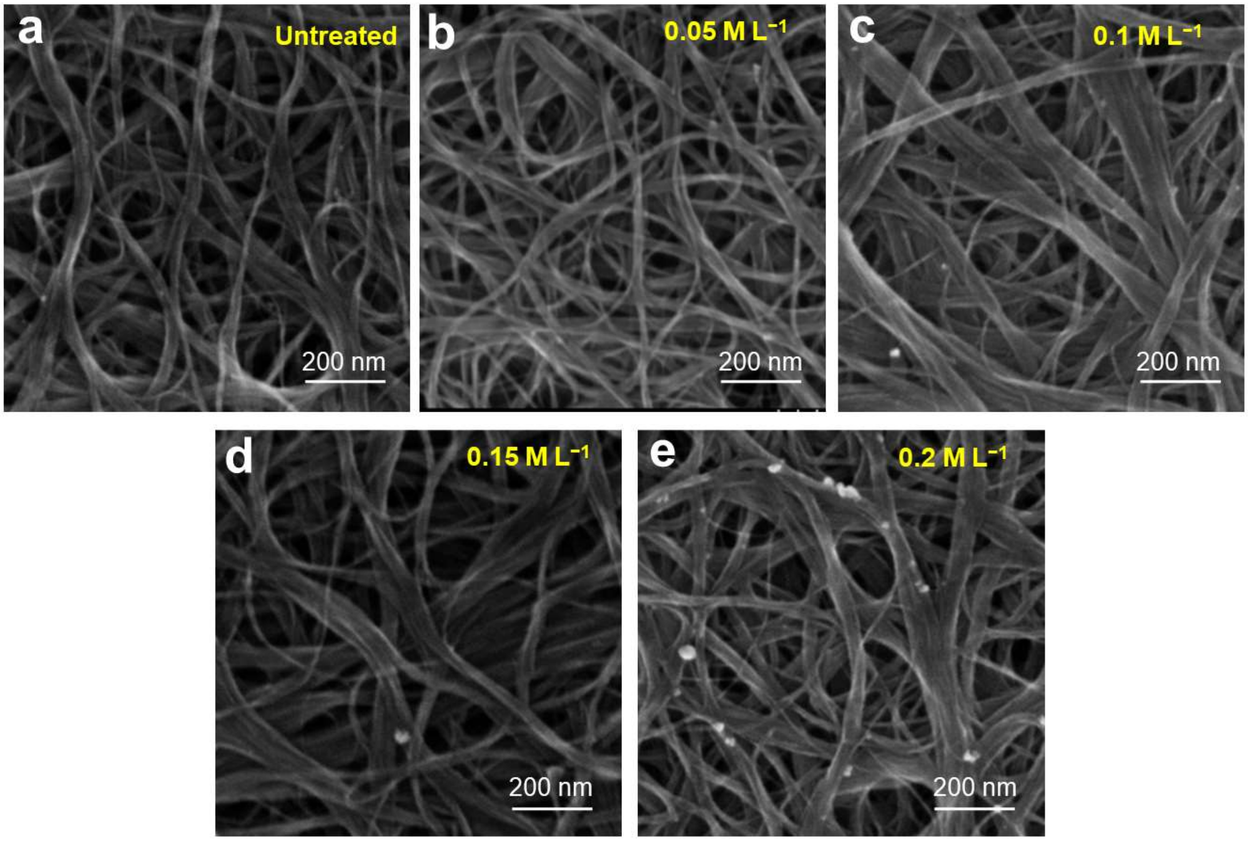

Supplementary Fig. 6 SEM images of SWCNT films by different concentrations of $NaBH_4$ treatments. **a** Untreated, and treated by (**b**) 0.05 M L^{-1}, (**c**) 0.1 M L^{-1}, (**d**) 0.15 M L^{-1}, and (**e**) 0.2 M L^{-1} $NaBH_4$ treatments. Here M is abbreviated from "mol".

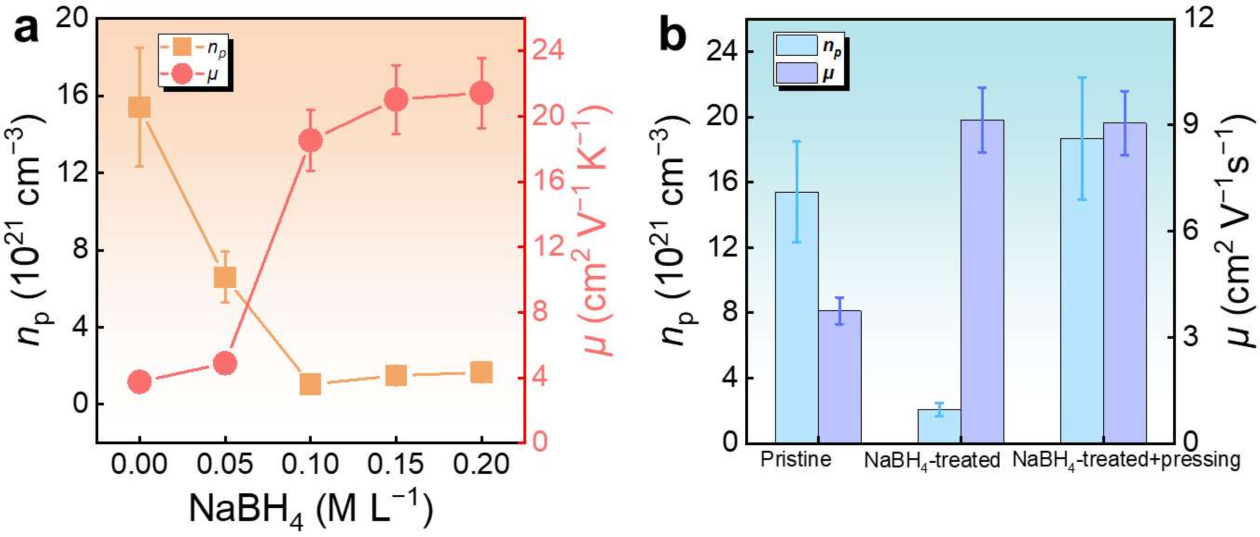

Supplementary Fig. 7 Measured carrier concentration n_p and mobility μ of the SWCNT films. Room-temperature n and μ (**a**) treated with different concentrations of $NaBH_4$ and (**b**) during triple treatments.

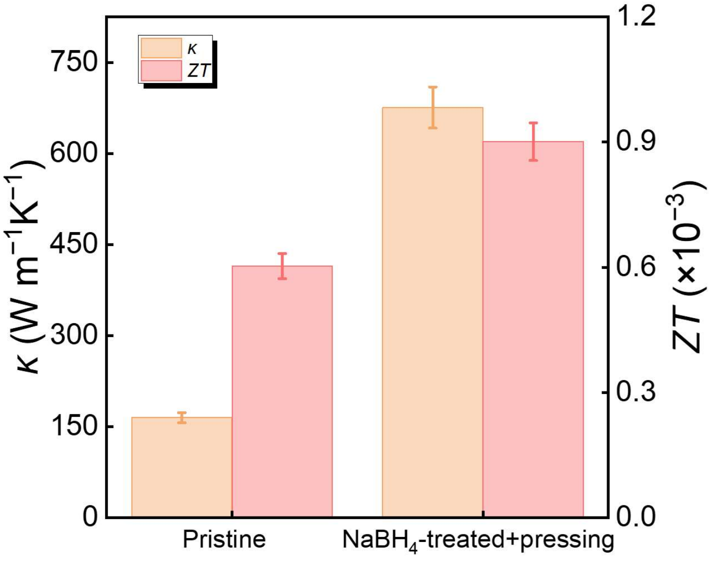

Supplementary Fig. 8 Thermal conductivity κ and *ZT* of untreated SWCNT film and 0.1 M L^{-1} $NaBH_4$-treated SWCNT film after cold-pressing.

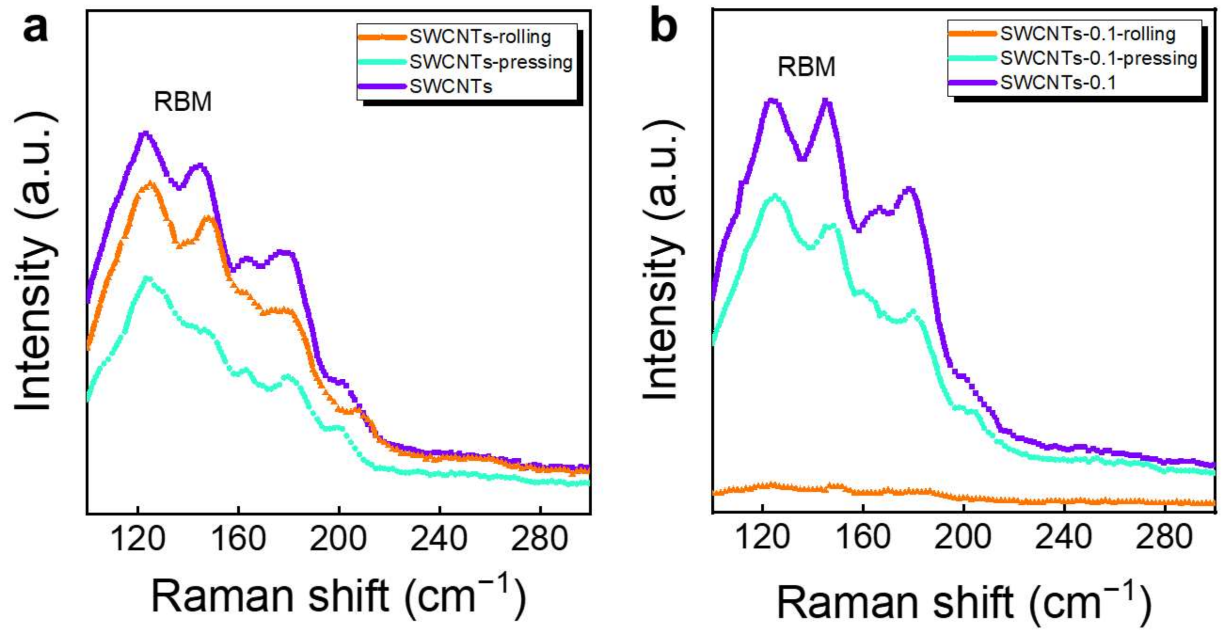

Supplementary Fig. 9 Raman spectra of SWCNT films during triple treatments. **a** untreated and (**b**) 0.1 M L^{-1} $NaBH_4$-treated pristine, cold-pressed, and rolled SWCNT films**.** Here RBM is abbreviated from radial breathing mode.

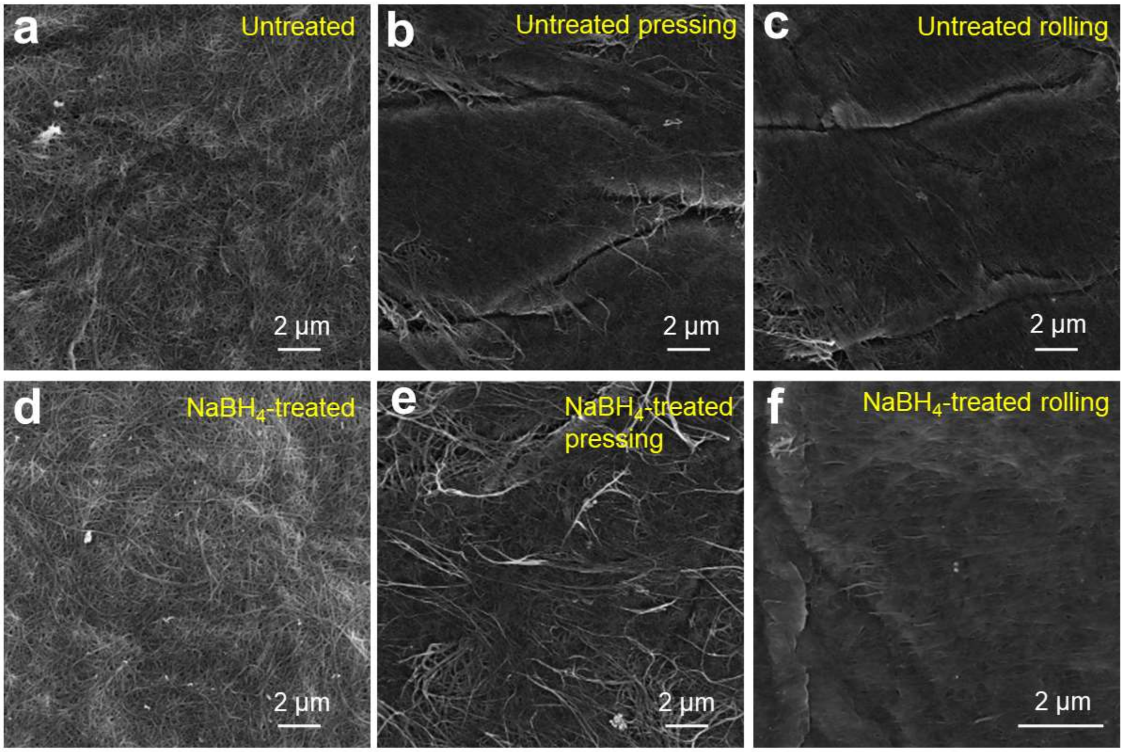

Supplementary Fig. 10 SEM images of SWCNT films by different treatments. SWCNT films without $NaBH_4$ treatment (**a**) before cold pressing or rolling, (**b**) after cold pressing, and (**c**) after rolling. SWCNT films with $NaBH_4$ treatment (**d**) before cold pressing or rolling, (**e**) after cold pressing, and (**f**) after rolling.

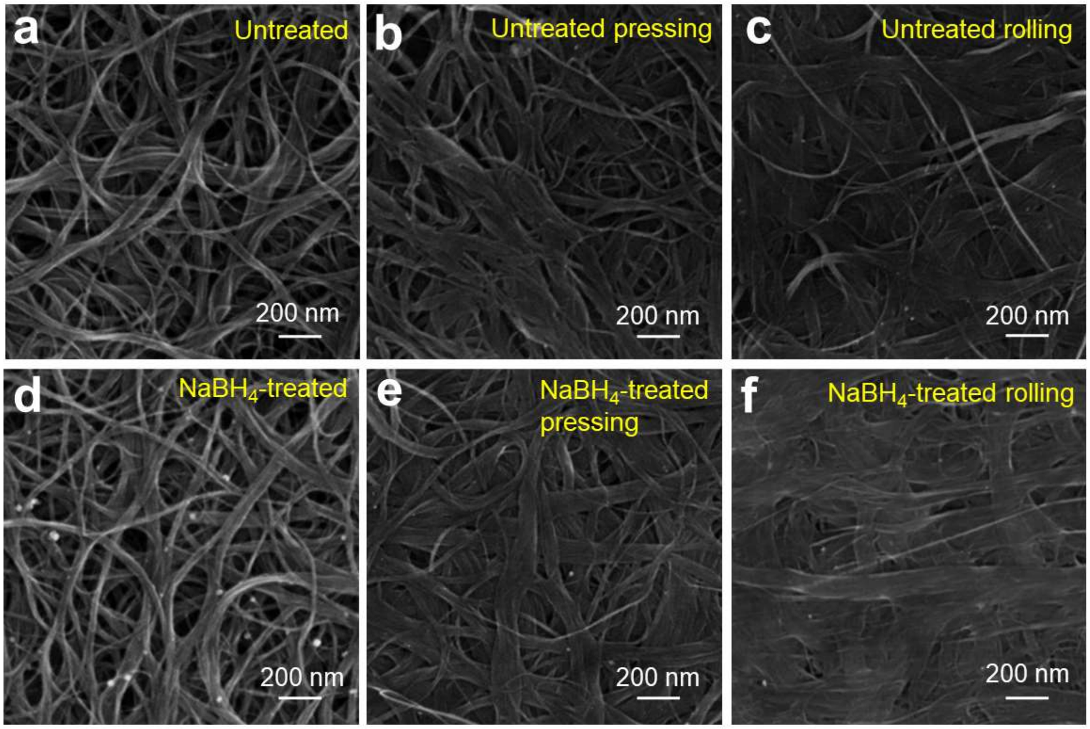

Supplementary Fig. 11 High-magnification SEM images of SWCNT films by different treatments. SWCNT films without $NaBH_4$ treatment (**a**) before cold pressing or rolling, (**b**) after cold pressing, and (**c**) after rolling. SWCNT films with $NaBH_4$ treatment (**d**) before cold pressing or rolling, (**e**) after cold pressing, and (**f**) after rolling.

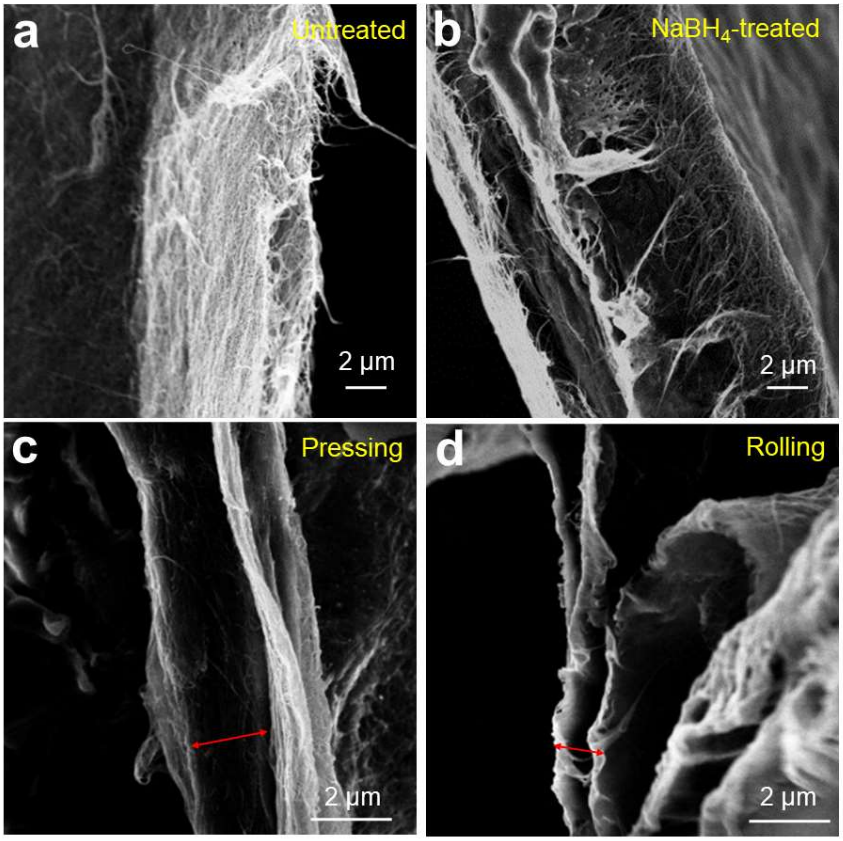

Supplementary Fig. 12 Cross-sectional SEM images of SWCNT films. **a** Untreated SWCNT films. **b** $NaBH_4$-treated SWCNT films. **c** cold-pressed SWCNT films with 0.1 M L^{-1} $NaBH_4$ treatment. **d** rolled SWCNT films with 0.1 M L^{-1} $NaBH_4$ treatment.

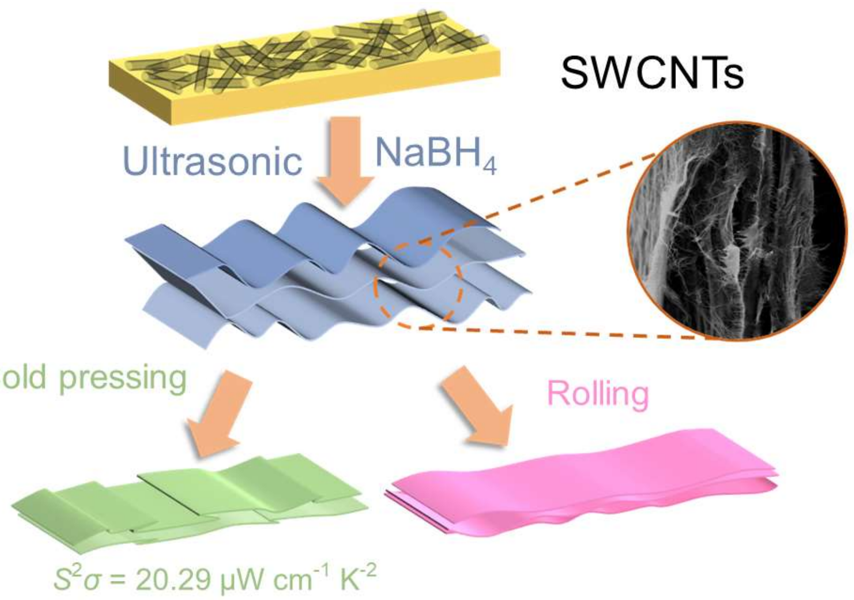

Supplementary Fig. 13 Illustration of the structures of the SWCNT films during triple treatments.

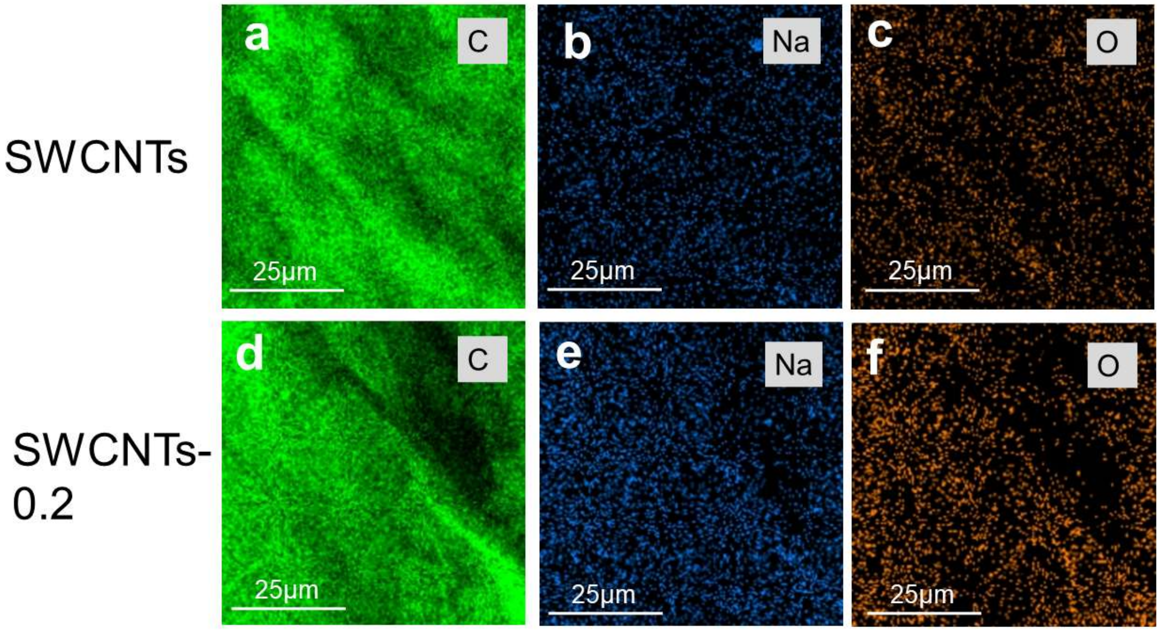

Supplementary Fig. 14 Energy-dispersive spectroscopy (EDS) maps of SWCNTs film with and without $NaBH_4$ treatment. **a** C, (**b**) Na and (**c**) O elements of untreated SWCNT films. **d** C, (**e**) Na and (**f**) O of 0.2 M L^{-1} $NaBH_4$-treated SWCNT films.

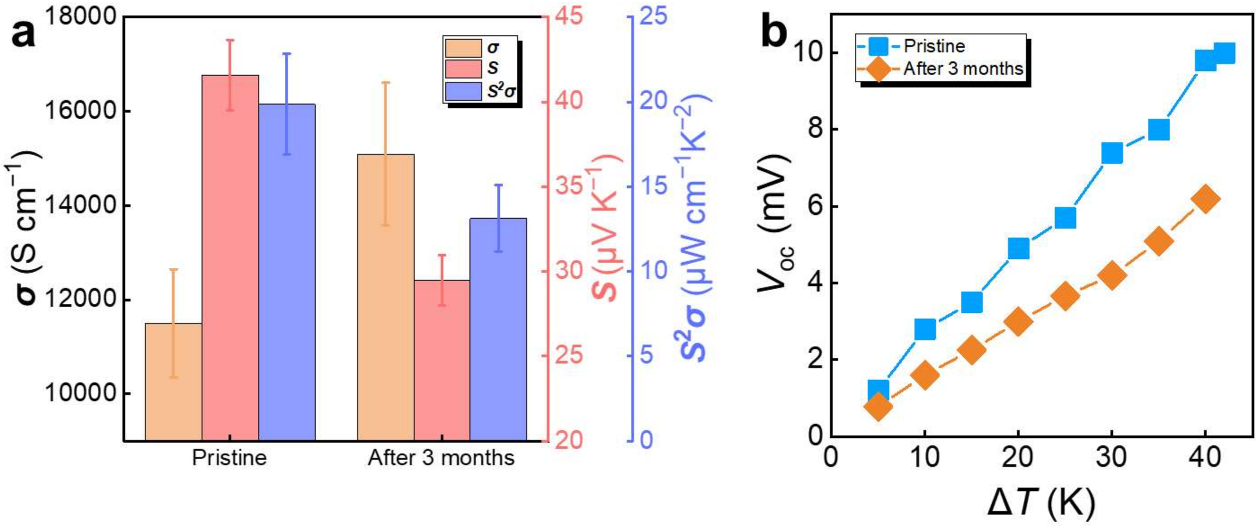

Supplementary Fig. 15 The air stability of the SWCNT films after triple treatments and the six-legged device after 3 months. **a** σ, S, and $S^2\sigma$ of SWCNT films with triple-treatments and (**b**) open-circuit voltage V_{oc} of the device as a function of temperature difference ΔT before and after being exposed to the air for three months.

References

1. Murrey, T.L. et al. Tuning counterion chemistry to reduce carrier localization in doped semiconducting carbon nanotube networks. *Cell Rep. Phys. Sci.* **4**, 101407 (2023).

2. Liu, S., Li, H. & He, C. Simultaneous enhancement of electrical conductivity and seebeck coefficient in organic thermoelectric SWNT/PEDOT:PSS nanocomposites. *Carbon* **149**, 25-32 (2019).

3. Zhang, M. et al. Highly electrical conductive PEDOT:PSS/SWCNT flexible thermoelectric films fabricated by a high-velocity non-solvent turbulent secondary doping approach. *ACS Appl. Mater. Interfaces* **15**, 10947-10957 (2023).

4. Nonoguchi, Y., Takata, A., Goto, C., Kitano, T. & Kawai, T. Thickness-dependent thermoelectric power factor of polymer-functionalized semiconducting carbon nanotube thin films. *Sci. Technol. Adv. Mat.* **19**, 581-587 (2018).

5. Zhang, L. et al. Achieving high thermoelectric properties in PEDOT:PSS/SWCNTs composite films by a combination of dimethyl sulfoxide doping and $NaBH_4$ dedoping. *Carbon* **196**, 718-726 (2022).

6. Fan, W., Guo, C.-Y. & Chen, G. Flexible films of poly(3,4-ethylenedioxythiophene)/carbon nanotube thermoelectric composites prepared by dynamic 3-phase interfacial electropolymerization and subsequent physical mixing. *J. Mater. Chem. A* **6**, 12275-12280 (2018).

7. Horike, S. et al. Bicyclic-ring base doping induces n-type conduction in carbon nanotubes with outstanding thermal stability in air. *Nat. Commun.* **13**, 3517 (2022).

8. Komoto, J., Goto, C., Kawai, T. & Nonoguchi, Y. Rational primary structure design for boosting the thermoelectric properties of semiconducting carbon nanotube networks. *Appl. Phys. Lett.* **118**, 261904 (2021).

9. Yin, S. et al. Enhancing thermoelectric performance of polyaniline/single-walled carbon nanotube composites *via* dimethyl sulfoxide-mediated electropolymerization. *ACS Appl. Mater. Interfaces* **13**, 3930-3936 (2021).

10. Nonoguchi, Y. et al. Simple salt-coordinated n-type nanocarbon materials stable in air. *Adv. Funct. Mater.* **26**, 3021-3028 (2016).

11. Fan, W., Liang, L., Zhang, B., Guo, C.-Y. & Chen, G. PEDOT thermoelectric composites with excellent power factors prepared by 3-phase interfacial electropolymerization and carbon nanotube chemical doping. *J. Mater. Chem. A* **7**, 13687-13694 (2019).

12. Kim, T.-H., Jang, J.G., Kim, S.H. & Hong, J.-I. Molecular engineering for enhanced thermoelectric performance of single-walled carbon nanotubes/π-conjugated organic small molecule hybrids. *Adv. Sci.* **10**, 2302922 (2023).

13. Zhou, W. et al. High-performance and compact-designed flexible thermoelectric modules enabled by a reticulate carbon nanotube architecture. *Nat. Commun.* **8**, 14886 (2017).

14. Song, H. et al. Polymer/carbon nanotube composite materials for flexible thermoelectric power generator. *Compos. Sci. Technol.* **153**, 71-83 (2017).

15. Wei, S., Zhang, Y., Lv, H., Deng, L. & Chen, G. SWCNT network evolution of PEDOT:PSS/SWCNT composites for thermoelectric application. *Chem. Eng. J.* **428**, 131137 (2022).

16. Lin, P.-S., Lin, J.-M., Tung, S.-H., Higashihara, T. & Liu, C.-L. Synergistic interactions in sequential process doping of polymer/single-walled carbon nanotube nanocomposites for enhanced n-type thermoelectric performance. *Small* **20**, 2306166 (2024).

17. Cao, G. et al. Simultaneously achieving green p- and n-type single-walled carbon nanotube composites by natural amino acids with high performance for thermoelectrics. *ACS Sustain. Chem. Eng.* **10**, 12009-12015 (2022).
